# Patient satisfaction with deep versus light/moderate sedation for non-surgical procedures: A systematic review and meta-analysis

**DOI:** 10.1097/MD.0000000000027176

**Published:** 2021-09-10

**Authors:** Hiroshi Hoshijima, Hitoshi Higuchi, Aiji Sato (Boku), Makiko Shibuya, Yoshinari Morimoto, Toshiaki Fujisawa, Kentaro Mizuta

**Affiliations:** a Division of Dento-oral Anesthesiology, Tohoku University Graduate School of Dentistry, Seiryomachi 1-1, Aoba-ku, Sendai, Japan; b Department of Anesthesiology, Saitama Medical University Hospital, Moroyama, Saitama, Japan; c Department of Dental Anesthesiology, Okayama University Hospital, 2-5-1 Shikata Kitaku, Okayama, Japan; d Department of Anesthesiology, Aichi Gakuin University School of Dentistry, 2-11 Suemori-dori, Chikusa-ku, Nagoya, Aichi, Japan; e Dental Anesthesiology, Department of Oral Pathobiological Science, Faculty of Dental Medicine and Graduate School of Dental Medicine, Hokkaido University, Kita-13, Nishi-7, Kita-ku, Sapporo, Japan; f Department of Critical Care Medicine and Dentistry, Graduate School of Dentistry, Kanagawa Dental University, 82, Inaoka-cho, Yokosuka, Kanagawa, Japan.

**Keywords:** adverse effect, deep sedation, meta-analysis, patient's satisfaction

## Abstract

**Background:**

Deep sedation relieves a patient's anxiety and stress during the procedure by inducing patient unconsciousness. However, it remains unclear whether deep sedation actually improves patient satisfaction with the procedure. Therefore, we performed a systematic review and meta-analysis to compare the satisfaction of patients undergoing deep sedation with that of those undergoing light/moderate sedation during non-surgical procedures.

**Methods:**

A comprehensive literature search was performed using electronic databases (search until September 2020). The primary outcome was whether patient satisfaction was higher after deep sedation or light/moderate sedation. The secondary outcome was the relative safety of deep sedation compared with light/moderate sedation in terms of oxygen saturation, systolic blood pressure, and heart rate. The tertiary outcomes were the relative procedure and recovery times for deep versus light/moderate sedation.

Data from each of the trials were combined, and calculations were made using DerSimonian and Laird random effects models. The pooled effect estimates for patient satisfaction were evaluated using relative risk (RR) with the 95% confidence interval (CI). The pooled effect estimates for continuous data are expressed as weighted mean difference with the 95% CI. We assessed heterogeneity with the Cochrane Q statistic and the I^2^ statistic. The risk of bias assessment and Grading of Recommendations Assessment, Development and Evaluation approach were used as the quality assessment method.

**Results:**

After removing unrelated studies and applying the exclusion criterion, 5 articles satisfied the inclusion criteria. Patient satisfaction was significantly higher in those who received deep sedation compared with light/moderate sedation (relative risk = 1.12; 95% CI, 1.04–1.20; *P* = .003; Cochrane Q = 25.0; I^2^ = 76%).

There was no significant difference in oxygen saturation, systolic blood pressure, heart rate, and procedure times according to whether the procedures were performed under deep or light/moderate sedation. However, the recovery time was significantly prolonged in patients under deep sedation.

**Conclusions:**

Our meta-analysis suggests that deep sedation resulted in improved patient satisfaction compared with light/moderate sedation. Deep sedation is recommended for patients undergoing procedures because it improves patient satisfaction. However, respiration and circulation should be carefully monitored both intra-operatively and postoperatively.

## 1 Introduction

According to the American Society of Anesthesiologists Guidelines for Sedation and Analgesia by Non-Anesthesiologists, sedation/analgesia has 2 important benefits. First, it allows patients to tolerate unpleasant procedures by relieving anxiety, discomfort, and pain. Second, in children and uncooperative adults, sedation/analgesia may shorten the time taken to perform procedures that are not particularly uncomfortable but require the patient not to move.^[1]^

Intravenous sedation and inhalation sedation have traditionally been used in surgery and endoscopic examinations for light to moderate sedation. However, light to moderate sedation may sometimes fail to sufficiently satisfy the patient because it is not possible to completely alleviate the patient's anxiety and pain.

In recent years, deep sedation has been used to improve patient satisfaction by reducing patient's anxiety and pain. According to the practice guidelines of the American Society of Anesthesiologists, the definition of deep sedation is “purposeful response after repeated or painful stimulation”, and the patient's level of consciousness is unconscious, which would be ideal for the patient during the procedure. Deep sedation relieves anxiety and stress during the procedure by inducing patient unconsciousness. However, it remains unclear whether deep sedation actually improves patient satisfaction with the procedure. In 2006, VanNatta and Rex^[2]^ compared patient satisfaction with colonoscopy using deep versus moderate sedation. They reported that deep sedation resulted in higher patient satisfaction with colonoscopy. However, studies by Paspatis et al^[3]^ and Allen et al^[4]^ reported that deep sedation does not improve patient satisfaction compared to light/moderate sedation. Furthermore, it remains unclear whether deep sedation or light/moderate sedation is superior in terms of vital signs, procedure time, and recovery time during procedures.^[2–6]^

In the present study, we performed a systematic review and meta-analysis of several randomized controlled trials to compare the satisfaction of patients undergoing deep sedation with the satisfaction of those undergoing light/moderate sedation during non-surgical procedures. The aim of this study was to examine patient satisfaction to determine whether deep sedation is superior to light/moderate sedation in non-surgical procedures. To assess secondary outcomes, we performed a systematic review and meta-analysis of oxygen saturation, blood pressure, and heart rate to compare deep sedation with light/moderate sedation. Finally, to assess tertiary outcomes, we compared whether procedure time and recovery time are different between deep sedation and light/moderate sedation.

## 2 Methods

This quantitative systematic review was performed according to the criteria outlined in the Preferred Reporting Items for Systematic Reviews and Meta-Analyses statement.^[7]^ First, we established the analysis methods and set the inclusion and exclusion criteria used in this meta-analysis, and then we registered the study protocol in the UMIN Clinical Trials Registry (registration number: UMIN 000032776).

### 2.1 Inclusion and exclusion criteria

The inclusion criteria were a prospective randomized study design, studies that compared deep, light or moderate sedation, studies that examined patient satisfaction, vital signs, procedure time, and recovery time. We also included studies that examined procedures not requiring tracheal intubation in adult patients. The exclusion criterion was a stay in the intensive care unit.

### 2.2 Search strategy

A comprehensive literature search was performed using MEDLINE, the Cochrane Central Register of Controlled Trials, EMBASE, and Scopus. The following strategy was devised for the PubMed search: (deep sedation“[MeSH Terms] OR (”deep“[All Fields] AND ”sedation“[All Fields]) OR ”deep sedation“[All Fields]) AND (Clinical Trial[ptyp] AND ”humans"[MeSH Terms]) (Supplemental Digital Content 1). A manual search of the references listed in the reports and reviews was also performed. There were no restrictions regarding the language of the article or publication type. The most recent search was performed in September 2020.

### 2.3 Selection of included studies

#### 2.3.1 Data extraction

Each article was independently assessed by authors HH and TF to determine whether the inclusion criteria were met. Disagreements in regard to values or analysis assignments were resolved through discussion. We attempted to avoid including data from any duplicate publications. We contacted the relevant author directly if we suspected any discrepancies in the data. Each author used a standardized data collection form to perform independent data abstraction.

The primary outcome of this systematic review was to determine whether deep sedation improved patient satisfaction compared to light/moderate sedation. To evaluate patient satisfaction, we extracted information from scoring evaluations of patient satisfaction after surgery. The patient satisfaction analyzed the number of people who compared the number of excellent or highly satisfied and others. The secondary outcome was to investigate whether deep sedation is safe compared to light/moderate sedation. Data extracted from eligible studies included values of oxygen saturation, systolic blood pressure, and heart rate. As the tertiary outcome, we compared procedure time and recovery time between deep sedation and light/moderate sedation.

Deep sedation was defined as a modified observer's alertness/sedation scale score < 2,^[2,3]^ Ramsay sedation scale score > 4,^[5]^ and bispectral index < 70,^[4]^ and observer assessment of alertness/sedation = 2.^[6]^ These definitions were according to the definitions determined for each randomized controlled trial including this meta-analysis.

### 2.4 Critical appraisal of study quality

#### 2.4.1 Risk of bias assessment

Risks of bias were estimated in the following methodological domains: sequence generation, allocation concealment, blinding of participants, incomplete outcome data, selective outcome reporting, and other potential threats to validity.^[8]^

#### 2.4.2 Quality of evidence assessment

The Grading of Recommendations Assessment, Development and Evaluation (GRADE) approach^[9]^ was used along with GRADEpro software (version 3.6 for Windows; available from http://ims.cochrane.org/revman/gradepro) to assess quality of evidence of the main outcomes.

### 2.5 Data synthesis and analysis

Data from each of the trials were combined, and calculations were made using DerSimonian and Laird random effects models. The pooled effect estimates for patient satisfaction were evaluated using relative risk (RR) with the 95% confidence interval (CI). The pooled effect estimates for continuous data (oxygen saturation, systolic blood pressure, heart rate, procedure time, and recovery time) are expressed as weighted mean difference (WMD) with the 95% CI. The Cochrane Q statistic and the I^2^ statistic, which indicates the percentage of variability due to heterogeneity rather than that due to sampling error, were used to test the homogeneity of the effect size across all trials.^[10]^

Publication bias often affects the validity of meta-analyses because studies showing no significant difference frequently go unpublished. Therefore we evaluated the potential for publication bias by generating a funnel plot by plotting RR values against the associated standard errors^[11]^ and using Begg test to assess the funnel plot's symmetry.^[12]^ Publication bias was considered present when the *P* value of the asymmetry test was <.1. However, we did not evaluate publication bias at all if the number of studies included in an analysis was <10. Review Manager (ver. 5.2, Nordic Cochrane Centre, The Cochrane Collaboration, Copenhagen, Denmark) was used for all statistical analyses.

## 3 Results

### 3.1 Characteristics of the included studies

**Figure 1:**
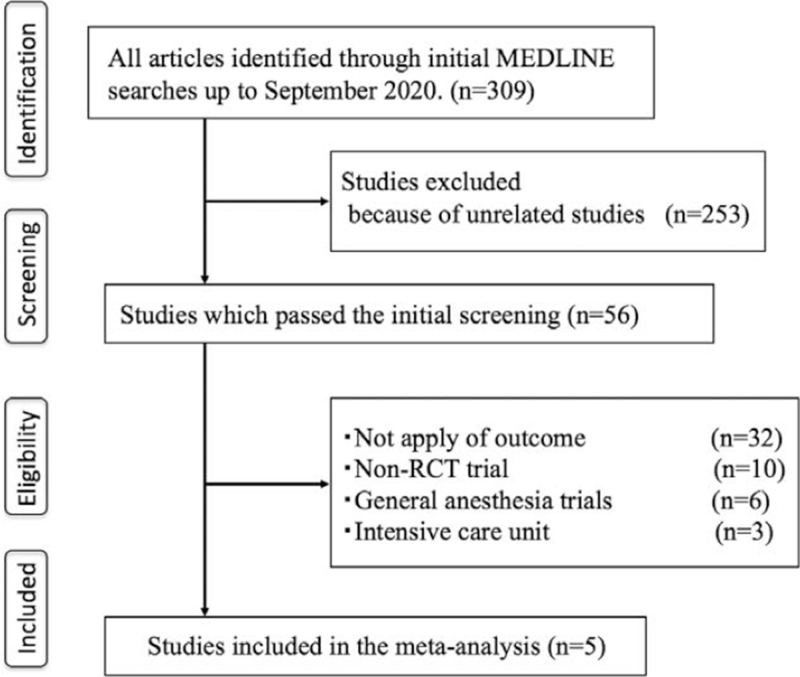
Flow diagram showing the literature search process. RCT = randomized controlled trial.

We identified 309 articles for review from the initial search of the electronic databases. We excluded 253 studies because they were unrelated to this research. We then thoroughly examined the remaining 56 articles to determine if the inclusion criteria were met. We excluded a further 51 studies because they did not report patient satisfaction results (n = 32), were not randomized controlled trials (n = 10) or studies using general anesthesia (n = 6), or were performed in an intensive care unit (n = 3). The full-text reading is shown in Supplemental Digital Content 2. The remaining 5 articles contained the necessary data for the planned comparison and met the inclusion criteria, as shown in Figure 1.^[2–6]^ Details of the selected trials are summarized in Table 1.

**Table 1:** Summary of including studies.

Author name	Year	Type of procedure	D/L, M (number)	Evaluation of depth of anesthesia	Sedative drugs	Definition of light/moderate sedation	Definition of deep sedation	Provider
VanNatta MG	2006	Colonoscopy	50/150	MOAAS/S	Deep sedation; propofol, Moderate sedation; propofol and fentanyl, or propofol and midazolam, or propofol and fentanyl and midazolam	MOAAS/S; median 3.2–4.0, mean 3.2–3.9	MOAAS/S; median 0.6, mean 0.9	Nurse
Paspatis GA	2011	Colonoscopy	258/262	MOAAS/S	Both sedation; midazolam with pethidine	MOAAS/S, 3	MOAAS/S < 2	Nurse
Lan C	2013	Upper gastrointestinal endoscopy	149/150	RSS	Deep sedation; propofol, remifentanil, and midazolam, Moderate sedation; N2O	RSS score, 3–4	RSS score > 4	Anesthesiologist
Allen M	2015	Colonoscopy	100/99	BIS	Both sedation; propofol	BIS, 70–80	BIS < 60	Anesthesiologist
Haga T	2016	Fiberoptic bronchoscopy	40/40	OASS	Deep and moderates sedation; midazolam, and internal codeine	OASS > 2	OASS, 2	Anesthesiologist

### 3.2 Results of the meta-analysis

#### 3.2.1 Primary outcome

**Figure 2:**
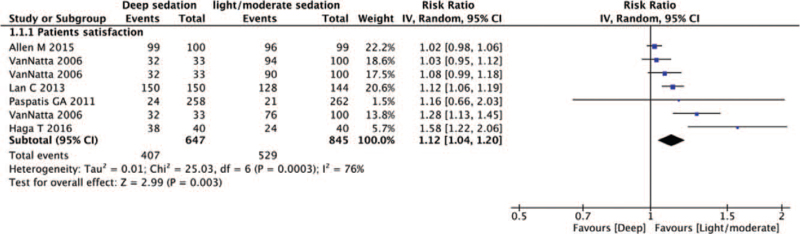
Forest plot of the patient satisfaction of deep sedation compared with the light/moderate sedation. The center of each square represents the relative risk for individual trials, and the corresponding horizontal line represents the 95% CI. The diamonds represent the pooled results. CI = confidence interval.

In the evaluation of patient satisfaction, 658 patients received deep sedation and 655 received light/moderate sedation. Meta-analysis of the 5 trials showed that patient satisfaction was higher after deep sedation than after light/moderate sedation. (RR = 1.12; 95% CI, 1.04–1.20; *P* = .003; Cochrane Q = 25.0; I^2^ = 76%) (Fig. 2).

#### 3.2.2 Secondary outcomes

**Figure 3:**
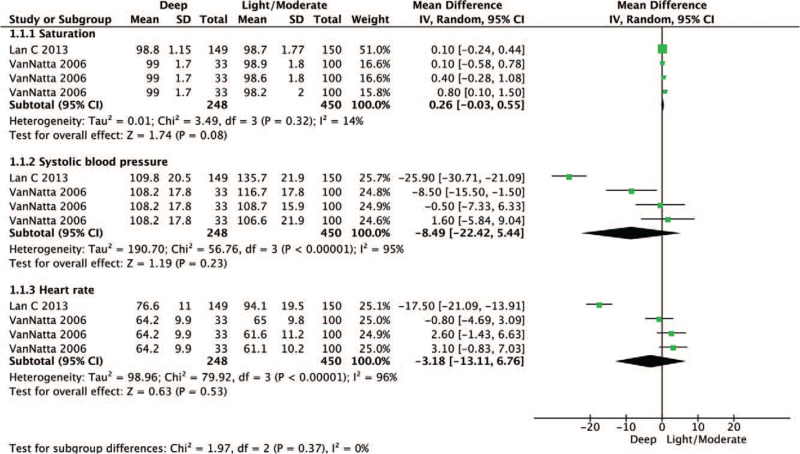
Forest plot of oxygen saturation, systolic blood pressure, and heart rate of deep sedation compared with the light/moderate sedation. The center of each square represents the weighted mean difference for individual trials, and the corresponding horizontal line represents the 95% CI. The diamonds represent the pooled results. CI = confidence interval.

We performed a systematic review and meta-analysis of oxygen saturation, systolic blood pressure, and heart rate values to compare deep sedation with light/moderate sedation as secondary outcomes. None of the 3 parameters were significantly different when comparing deep sedation to light/moderate sedation (oxygen saturation: WMD = 0.26, 95% CI –0.03 to 0.55, *P* = .08; Cochrane Q = 3.49, I^2^ = 14%; systolic blood pressure: WMD = –8.49, 95% CI –22.4 to 5.44, *P* = .23, Cochrane Q = 56.7, I^2^ = 95%; and heart rate: WMD = –3.18, 95% CI –13.1 to 6.78, *P* = 0.53, Cochrane Q = 79.9, I^2^ = 96%) (Fig. 3).

#### 3.2.3 Tertiary outcomes

**Figure 4:**
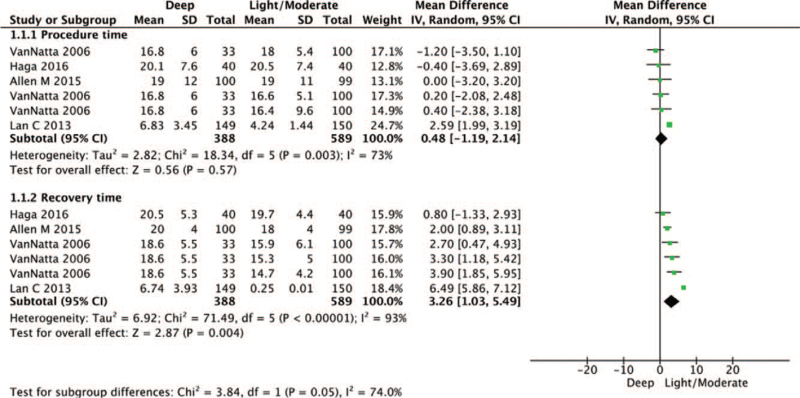
Forest plot of procedure time and recovery time between deep sedation and light/moderate sedation. The center of each square represents the weighted mean difference for individual trials, and the corresponding horizontal line represents the 95% CI. The diamonds represent the pooled results. CI = confidence interval.

We compared procedure time and recovery time between deep sedation and light/moderate sedation as tertiary outcomes. Procedure times were not significantly different between deep sedation and light/moderate sedation (WMD = 0.48, 95% CI –1.19 to 2.14, *P* = .57, Cochrane Q = 18.3, I^2^ = 73%). However, recovery time was significantly prolonged in patients undergoing deep sedation versus light/moderate sedation (WMD = 3.26, 95% CI 1.03–5.49, *P* = .004, Cochrane Q = 71.5, I^2^ = 93%) (Fig. 4).

### 3.3 Quality of evidence

**Figure 5:**
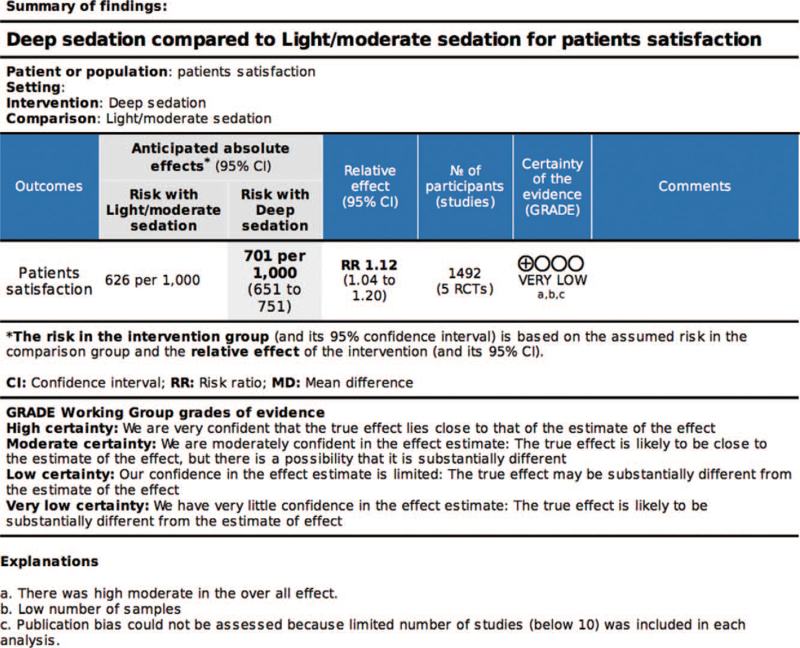
The Grading of Recommendations Assessment, Development and Evaluation (GRADE) approach.

The quality of evidence in terms of patient satisfaction was graded as very low for deep sedation compared with that for light/moderate sedation. The articles included in this comparison had a moderate heterogeneity. Moreover, small-study effects could not be assessed using funnel plots because less than 10 studies were analyzed (Fig. 5).

### 3.4 Risk of bias assessment

**Figure 6:**
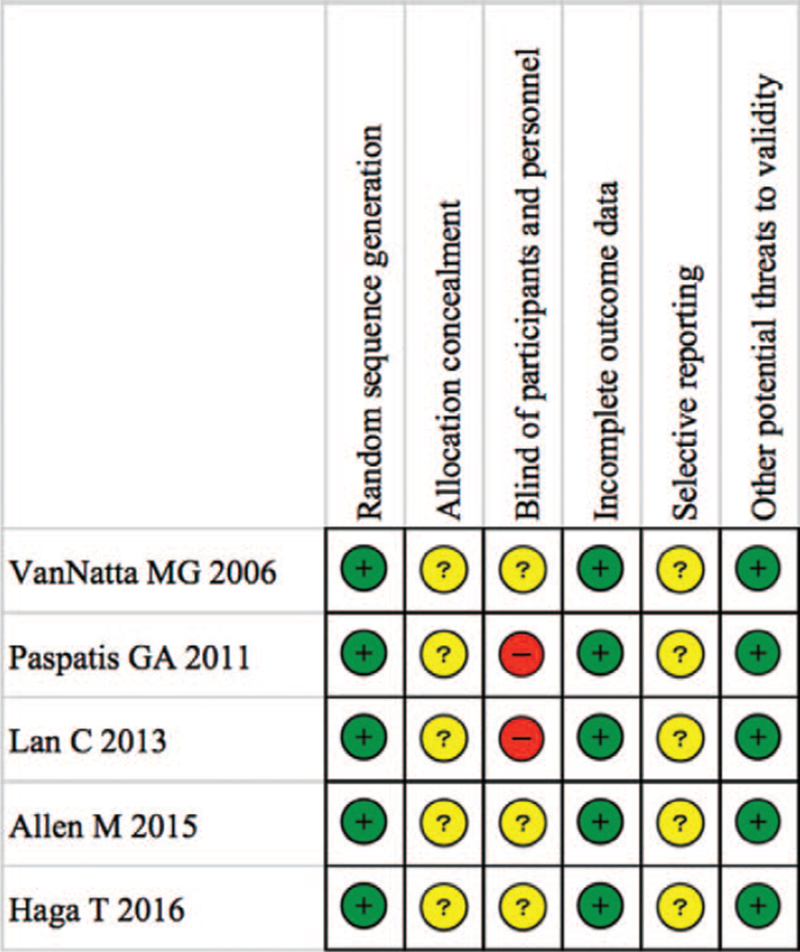
Risk of bias. Green circles, red circles, and yellow circles indicate “low risk of bias”, “high risk of bias”, and “unclear risk of bias”, respectively.

In the assessment of risk of bias, some random sequence generation was performed in all studies. However, the allocation concealment could not be confirmed. Also, all studies failed to confirm pre-registration of the study, increasing the risk of selective reporting. Lack of allocation concealment and selective reporting is a factor in raising bias. The risks of bias are summarized in Figure 6.

### 3.5 Publication bias

Publication bias was not evaluated because the number of studies included in the analysis was small (<10).

## 4 Discussion

This study reveals that deep sedation improved patient satisfaction compared with light/moderate sedation. However, recovery time was significantly prolonged in patients undergoing deep sedation compared with those undergoing light/moderate sedation. There were no significant differences in the values of oxygen saturation, systolic blood pressure, and heart rate between deep sedation and light/moderate sedation.

Deep sedation improves patient satisfaction by completely or largely rendering the patient unconscious during the procedure. VanNatta and Rex^[2]^ reported that 96% of patients in the deep sedation group did not wake during the procedure compared with 50% of patients who woke in the moderate sedation group. In addition, 14% of the patients felt pain in the moderate sedation group, whereas only 2% of patients reported pain in the deep sedation group.^[2]^ Therefore, loss of consciousness is considered to be a factor that improves patient satisfaction by preventing patients from feeling pain. Also, the lower incidence of recall may improve patient satisfaction. Allen et al^[4]^ reported that only 1% of patients experienced recall in the deep sedation group compared with 12% of patients in the moderate sedation group. Drugs with strong amnestic action may be effective in improving patient satisfaction. Shin et al^[13]^ reported that the group with high patient satisfaction with sedation had a significantly higher number of patients who experienced amnestic effects. Taylor et al^[14]^ also reported that pre-medication with intravenous midazolam 2 mg produced increased sedation, amnesia, and anxiolysis.

There were no significant differences in the values of oxygen saturation, systolic blood pressure, and heart rate between deep sedation and light/moderate sedation. In general, the probability that an adverse event such as desaturation, hypotension, or bradycardia occurs increases as the depth of anesthesia increases. In a recent study, Koers et al^[15]^ reported that desaturation occurred in 4.6%, hypotension occurred in 2.8%, and bradycardia occurred in 0.4% of patients who underwent moderate to deep sedation. These adverse events especially desaturation must be resolved immediately if they occur because desaturation can quickly threaten a patient's life. Its occurrence is often preventable, and even if it does occur, it is generally resolved before it becomes critical. In this way, even in the case of deep sedation, prevention and treatment are performed such that excessive desaturation and circulatory suppression do not occur due to strict respiratory and circulatory management by nurses and anesthesiologists. Deep sedation seems to cause a greater reduction in oxygen saturation, but the difference does not appear to be statistically significant. Moreover, only a relatively small number of studies and patients were analyzed, so the statistical power may have been inadequate to detect small yet clinically important differences between methods in the study outcomes. Additional research is needed to further evaluate the adverse effects of deep sedation in comparison with those of light/moderate sedation.

In this meta-analysis, recovery time was significantly longer after deep sedation than after light/moderate sedation. Generally, the greater the depth of anesthesia, the longer the time required to awake from anesthesia. In this analysis, the time to wake up following deep sedation was extended by about 4 minutes on average, which may not be clinically problematic. However, careful postoperative management is required in patients who receive deep sedation because of the prolonged recovery time.

### 4.1 Limitations

This research study has several limitations. The first is the limitations inherent in all meta-analyses as a result of the heterogeneity in design of the included studies. The second limitation is the inclusion of studies that used different types of sedative drugs, which may have introduced a degree of bias and lowered the quality of evidence. Third, the patients were not blinded to the protocol used. Fourth, differences in patient populations, concentrations of the sedative or analgesic drugs used, the procedures performed, the anesthesia provider, and the definition of deep sedation increase the heterogeneity further. All of these factors can create significant bias in the results of any meta-analysis. The magnitude of the differences between deep and moderate/light sedation on patient satisfaction was very minor (RR = 1.12) in our meta-analysis. This result may indicate that deep sedation may improve patient satisfaction but further research is needed.

## 5 Conclusions

The results of our meta-analysis suggest that deep sedation improved patient satisfaction compared with that for light/moderate sedation (GRADE: very low). Use of deep sedation is recommended because it improves patient satisfaction. However, respiration and circulation should be carefully monitored both intra-operatively and postoperatively. Furthermore, deep sedation requires a prolonged observation period because the postoperative recovery time is longer than that after light/moderate sedation.

## Author contributions

**Conceptualization:** Hiroshi Hoshijima.

**Data curation:** Hitoshi Higuchi.

**Investigation:** Makiko Shibuya, Yoshinari Morimoto, Toshiaki Fujisawa.

**Methodology:** Aiji Sato (Boku), Makiko Shibuya, Yoshinari Morimoto, Toshiaki Fujisawa.

**Supervision:** Aiji Sato (Boku), Kentaro Mizuta.

**Writing – original draft:** Hiroshi Hoshijima, Kentaro Mizuta.

**Writing – review & editing:** Hitoshi Higuchi.

## Supplementary Material

Supplemental Digital Content

## Supplementary Material

Supplemental Digital Content
