# Supplementary material for: Patient satisfaction with deep versus light/moderate sedation for non-surgical procedures: A systematic review and meta-analysis
Source: Medicine (Baltimore). 2021 Sep 10;100(36):e27176. doi: 10.1097/MD.0000000000027176 (PMC8428728; doi:10.1097/MD.0000000000027176)
Supplement: Supplemental Digital Content [file medi-100-e27176-s001.docx]

Supple 1.

***Search strategy for the Cochrane Central Register of Controlled Trials***

‘deep sedation or moderate sedation or light sedation or conscious sedation or "deep-sedation" or "moderate-sedation" or "light-sedation" or "conscious-sedation" or "satisfaction" or " patient satisfaction" or " patients satisfaction " or "hemodynamic response" or "hemodynamic change" in Title, Abstract, Keywords and operation or emergent or urgent in Title, Abstract, Keywords'

***Search strategy for Embase***

('deep sedation':ab,ti OR 'moderate sedation':ab,ti OR 'light sedation':ab,ti OR 'conscious sedation':ab,ti OR deep-sedation:ab,ti OR moderate-sedation:ab,ti OR light-sedation:ab,ti OR conscious-sedation:ab,ti) AND (atisfaction *:ab,ti,kw OR patient satisfaction *:ab,kw,ti) AND ('clinical article'/de OR 'clinical trial'/de OR 'comparative study'/de OR 'controlled clinical trial'/de OR 'controlled study'/de OR 'crossover procedure'/de OR 'human'/de OR 'human experiment'/de OR 'intermethod comparison'/de OR 'major clinical study'/de OR 'multicenter study'/de OR 'prospective study'/de OR 'randomized controlled trial'/de OR 'randomized controlled trial (topic)'/de)

**Scopus**

( TITLE-ABS-KEY ( deep AND sedation ) OR TITLE-ABS-KEY ( moderate AND sedation ) AND TITLE-ABS-KEY ( light AND sedation ) AND TITLE-ABS-KEY ( randomized ) AND NOT TITLE-ABS-KEY ( retrospective ) )
