# Supplementary material for: Patient satisfaction with deep versus light/moderate sedation for non-surgical procedures: A systematic review and meta-analysis
Source: Medicine (Baltimore). 2021 Sep 10;100(36):e27176. doi: 10.1097/MD.0000000000027176 (PMC8428728; doi:10.1097/MD.0000000000027176)
Supplement: Supplemental Digital Content [file medi-100-e27176-s002.docx]

Supplemental File 2

1. Kramer KJ, Ganzberg S, Prior S, Rashid RG: Comparison of propofol-remifentanil versus propofol-ketamine deep sedation for third molar surgery. Anesth Prog 59:107, 2012

2. Hagiwara A, Matsuura N, Ichinohe T: Comparison of Changes in Respiratory Dynamics Immediately After the Start of Propofol Sedation With or Without Midazolam. J Oral Maxillofac Surg 76:52, 2018

3. Dag C, Bezgin T, Ozalp N, Golcuklu Aydin G: Utility of bispectral index monitoring during deep sedation in pediatric dental patients. J Clin Pediatr Dent 39:68, 2014

4. Koers L, Eberl S, Cappon A, et al: Safety of moderate-to-deep sedation performed by sedation practitioners: A national prospective observational study. Eur J Anaesthesiol 35:659, 2018

5. Li Q, Zhou Q, Xiao W, Zhou H: Determination of the appropriate propofol infusion rate for outpatient upper gastrointestinal endoscopy-a randomized prospective study. BMC Gastroenterol 16:49, 2016

6. Schick A, Driver B, Moore JC, et al: Randomized Clinical Trial Comparing Procedural Amnesia and Respiratory Depression Between Moderate and Deep Sedation With Propofol in the Emergency Department. Acad Emerg Med 26:364, 2019

7. Klare P, Hartrampf B, Haller B, et al: Magnetic endoscope imaging for routine colonoscopy: impact on propofol dosage and patient safety - a randomized trial. Endoscopy 48:916, 2016
